# Supplementary material for: Does improved interpreter uptake reduce self-discharge rates in hospitalised patients? A successful hospital intervention explained
Source: PLoS One. 2021 Oct 12;16(10):e0257825. doi: 10.1371/journal.pone.0257825 (PMC8509875; doi:10.1371/journal.pone.0257825)
Supplement: S1 File — (DOCX) [file pone.0257825.s001.docx]

**Does improved interpreter uptake reduce self-discharge rates in hospitalised patients who speak diverse languages? A successful hospital intervention explained**

**Supplementary file (S1 file): Regression analysis**

Linear regression analysis was undertaken in Stata 15.1 to the examine the relationship between quarterly numbers of Aboriginal interpreter bookings and reported episodes of care ending discharge for Aboriginal patients. The ‘robust’ option was used to control for heteroskedasticity.

regress pct_tol quarterly_interp_bookings, robust

Linear regression Number of obs = 12

F(1, 10) = 7.74

Prob > F = 0.0194

R-squared = 0.4260

Root MSE = .62421

-----------------------------------------------------------------------------------------

| Robust

pct_tol | Coef. Std. Err. t P>|t| [95% Conf. Interval]

--------------------------+--------------------------------------------------------------

quarterly_interp_bookings | -.0078402 .0028187 -2.78 0.019 -.0141207 -.0015598

_cons | 13.9885 .8335761 16.78 0.000 12.13118 15.84582

-----------------------------------------------------------------------------------------

**Interpretation**

pct_tol: percentage of Aboriginal patients who took own leave (self discharged / discharged against medical advice) per quarter

quarterly_interp_bookings: number of Aboriginal interpreter bookings made per quarter

The beta coefficient was -0.0078

The p value was 0.019

The goodness of fit as indicated by R squared was 0.4260

**Post-estimation diagnostics** using predicted pct_tol plotted against actual pct_tol revealed an approximately directly proportional relationship. We plotted residuals against predicted values and did not observe any pattern, indicating appropriateness of the model.
