# Supplementary material for: Does improved interpreter uptake reduce self-discharge rates in hospitalised patients? A successful hospital intervention explained
Source: PLoS One. 2021 Oct 12;16(10):e0257825. doi: 10.1371/journal.pone.0257825 (PMC8509875; doi:10.1371/journal.pone.0257825)
Supplement: Attachment — Submitted filename: Review article March 2021.docx [file pone.0257825.s002.docx]

Review article March 2021

Does improved interpreter uptake reduce self-discharge rates in hospitalised patients? A successful hospital intervention explained

- In the data and definitions section ;

RDH 77 separations data, used as a measure of inpatient healthcare utilisation, were obtained for all 78 Indigenous people for the same timeframe.’

Previously you have used Aboriginal. Is Indigenous used here because of the hospitals own coding preference ? Otherwise I would suggest a consistent term be used throughout

- Could you make it more clear who the falls into the category of ‘clinicians’ ? is it doctors only or nurses and allied health also – if these parties were not trained is there a reason?
- Why was only surgical and emergency doctors trained , rather than other wards or specialities?
- The cultural safety paragraph is well written , clear and well referenced.

In this section you have included explanation on your terminology but I wonder if this concept would be better introduced earlier.

- ‘Additionally, implementation and delivery included Aboriginal leadership.’ Could you expand on this – who was consulted for the development of the training or the training of the interpreter coordinator, were these hospital employees? Doctors? Community members?
- Intervention component 3- you mentioned meeting with the champions about the barriers and facilitators – could you expand on what these were?
- ‘While the role was conceived as being a coordinator role to improve efficiency and ease of bookings, it was in fact realised somewhat differently, focussing more on Aboriginal staff support particularly, provision of mentoring for interpreters on assignment to the hospital, including helping them to navigate the hospital environment.’ This is interesting, I would like to know more about the training of the interpreters themselves, perhaps it is out of the scope of this article.
- It is good that you reported on the additional interventions occurring in the study period. It may be devaluing and overzealous to say that of these initiatives that it is ‘unlikely to have had a large role’ – perhaps alter your wording here. Also in regards to intern orientation – was there any other changes with cultural safety training from year to year – potentially the cultural safety knowledge overall was increased hence the appreciation of the interpreter service.
- The first paragraph of your discussion is very well worded, and very clear.
- Overall this paper is very clear and very well written.

Your table outlines some of the more specific details that I have questioned above but consider if there is scope to have some of your explanations expanded in the main body of the text. You’re reporting on the self discharge numbers and associations with interpreter use is quiet brief however I see this as the main focus of the paper – I am not sure if there is any date or scope to expand on this.

Otherwise I wonder if you could make any recommendations in your discussion about how this intervention might be applicable to other hospital or health service settings. I appreciate you’re suggestions for further research in this area.

Well done. I hope these interventions can continue to the be the ‘norm’ in the hospital.
