# Supplementary material for: Does improved interpreter uptake reduce self-discharge rates in hospitalised patients? A successful hospital intervention explained
Source: PLoS One. 2021 Oct 12;16(10):e0257825. doi: 10.1371/journal.pone.0257825 (PMC8509875; doi:10.1371/journal.pone.0257825)
Supplement: Attachment — Submitted filename: Response letter 2 PONE-D-21-06717_R2.docx [file pone.0257825.s004.docx]

20 July 2021

Dear Editor,

**Re: Second response to reviewer comments**

**PONE-D-21-06717 R1: Does improved interpreter uptake reduce self-discharge rates in hospitalised patients? A successful hospital intervention explained**

We thank both reviewers for re-reviewing. We also thank the editor for feedback about the discordance in the titles, the need for a clean manuscript file not including track changes, and the need for extra information in the financial disclosure. These have all been attended to. Our responses to the reviewers are as follows in bold font:

Reviewer #1: Thank you for your considered and detailed responses, the additional information provided and included will allow a better ease of readership and maintains focus on the titled issue of interpreters and impact on self discharge. I would encourage you to publish your future findings about interpreter training and support you have mentioned. Well done in your manuscript.

Reviewer #2: although all responses to reviewer 2 have been addressed, I have a couple of comments about the authors' responses to reviewer 1's questions.

1. Line 158, authors have added "mostly doctors, plus one nurse". - are you able to specify the number of doctors

**The text has now been updated to include the number provided in table 2: 126 doctors and 1 nurse.**

2. line 120, authors have responded to query about "aboriginal leadership" in their response to the reviewer but not changed anything in text - can you change to state that the Aboriginal Interpreter coordinator was involved in the implementation and delivery of the program - this removes any ambiguity around this

**We have revised this to read: ‘Additionally, implementation and delivery included Aboriginal leadership, provided through the Aboriginal Interpreter Coordinator, the Royal Darwin Hospital Aboriginal Support Unit and the Aboriginal Interpreter Service.’**

**This ensures all three Aboriginal authors who contributed to implementation and delivery of the study are adequately acknowledged.**

We greatly thank the reviewers for their helpful comments which have improved manuscript.

Yours sincerely,

Professor Anna Ralph (corresponding author)
